# Comparative effect of clopidogrel and aspirin versus aspirin alone on laboratory parameters: a retrospective, observational, cohort study

**DOI:** 10.1186/1475-2840-12-87

**Published:** 2013-06-14

**Authors:** Yasuo Takahashi, Yayoi Nishida, Tomohiro Nakayama, Satoshi Asai

**Affiliations:** 1 Division of Genomic Epidemiology and Clinical Trials, Clinical Trials Research Center, Nihon University School of Medicine, 30-1 Oyaguchi-Kami Machi, Itabashi-ku, Tokyo 173-8610, Japan; 2 Department of Pathology and Microbiology, Division of Laboratory Medicine, Nihon University School of Medicine, 30-1 Oyaguchi-Kami Machi, Itabashi-ku, Tokyo 173-8610, Japan; 3 Department of Biomedical Sciences, Division of Pharmacology, Nihon University School of Medicine, 30-1 Oyaguchi-Kami Machi, Itabashi-ku, Tokyo 173-8610, Japan

**Keywords:** Clopidogrel, Aspirin, Laboratory parameter, Antiplatelet therapy, Propensity-score adjustment

## Abstract

**Background:**

Clopidogrel and aspirin are antiplatelet agents that are recommended to reduce the risk of recurrent stroke and other cardiovascular events. Combination therapy of clopidogrel and aspirin has been shown to increase the risk of hemorrhage, but the effects of the drugs on laboratory parameters have not been well studied in patients in routine clinical practice. Therefore, we evaluated and compared the effects of combination therapy with clopidogrel plus aspirin and aspirin monotherapy on laboratory parameters using a clinical database.

**Methods:**

We used data from the Clinical Data Warehouse of Nihon University School of Medicine obtained between November 2004 and April 2011, to identify cohorts of new users (n = 159) of clopidogrel (75 mg/day) plus aspirin (100 mg/day) and new users (n = 834) of aspirin alone (100 mg/day). We used a multivariable regression model and regression adjustment with the propensity score to adjust for differences in baseline covariates between settings, and compare the mean changes in serum levels of creatinine, aspartate aminotransferase, alanine aminotransferase and hematological parameters, including hemoglobin level, hematocrit, and white blood cell (WBC), red blood cell and platelet counts up to two months after the start of study drug administration.

**Results:**

After adjustment, the reduction of WBC count in clopidogrel plus aspirin users was significantly greater than that in aspirin alone users. All other tests showed no statistically significant difference in the mean change from baseline to during the exposure period between clopidogrel plus aspirin users and aspirin alone users. The combination of clopidogrel and aspirin increased the risk of gastrointestinal bleeding compared with aspirin alone, with a relative risk ranging from 2.06 (95% CI, 1.02 to 4.13; p = 0.043) for the multivariate model and 2.61 (95% CI, 1.18 to 5.80; p = 0.0184) for propensity adjustment.

**Conclusion:**

Our findings suggested that hematological adverse effects may be greater with combination therapy of clopidogrel plus aspirin than with aspirin monotherapy.

## Background

Clopidogrel and aspirin are antiplatelet agents that are recommended to reduce the risk of recurrent stroke and other cardiovascular events [1,2]. Combination therapy, typically with clopidogrel and aspirin, is commonly used for the prevention of cardiovascular events, when given for an appropriate indication and duration [3]. Aspirin inhibits platelet cyclooxygenase by irreversible acetylation, thereby preventing the formation of thromboxane A2 which is a powerful stimulant of platelet aggregation [4]. Clopidogrel, a thienopyridine, acts by inhibiting adenosine receptors, which inhibits the early step of platelet activation [5]. Thus, the effect of combining aspirin and clopidogrel is synergistic in preventing platelet aggregation, and this combination may offer certain theoretical benefits over either agent alone. Some clinical trials have investigated the efficacy of the combination of clopidogrel and aspirin. The combination has been shown to reduce the risk of ischemic events in patients with myocardial infarction with or without ST-segment elevation, and after angioplasty or stenting [6-10]. On the other hand, in the MATCH trial, combination therapy with clopidogrel plus aspirin in patients with a prior stroke or TIA showed no significant benefit in reducing major vascular events compared with clopidogrel alone [11]. The CHARISMA trial has shown that the combination of clopidogrel and aspirin was not significantly more effective than aspirin alone in reducing the rate of myocardial infarction, stroke, or death from cardiovascular causes [12]. Based on these clinical findings, combination therapy with aspirin and clopidogrel is recommended for treatment of acute coronary syndromes and the prevention of coronary events after placement of a stent as the most appropriate indications [1]. The nature of antiplatelet therapy involves an inherent risk of bleeding complications. Although this combination of antiplatelet agents has been demonstrated to offer clinical benefits under certain conditions, it does raise the risk of bleeding complications [13]. Thus, there is a consensus that dual antiplatelet therapy involves the issue of bleeding risk. Although previous reports have assessed the side effects of antiplatelet agents, they usually focused on the adverse events of antiplatelet agents [14,15], and few studies have focused on the effects of the drugs on laboratory parameters. Therefore, we evaluated and compared the effects of combination therapy with clopidogrel plus aspirin and aspirin monotherapy on laboratory parameters including creatinine, aspartate aminotransferase (AST), and alanine aminotransferase (ALT) levels and hematological parameters including red blood cell (RBC) count, white blood cell (WBC) count, platelet count, hemoglobin level and hematocrit, which are typically used in clinical practice for checking side effects of drugs.

## Methods

### Data source

We obtained the study data from electronic medical records stored in the Nihon University School of Medicine (NUSM) Clinical Data Warehouse (CDW), which is a centralized data repository that integrates separate databases, including an order entry database and a laboratory results database, from the hospital information systems at three hospitals affiliated with NUSM, and is described elsewhere [16]. The prescription database in the CDW contains information from approximately 0.6 million patients, and prescribing data are linked longitudinally to detailed clinical information such as patient demographics, diagnosis, and laboratory data. Several epidemiological studies examining the effects of drugs on laboratory parameters using NUSM’s CDW have been published [17-20].

### Study population

The cohorts identified for the study included Japanese patients aged over 20 years who had been newly treated with clopidogrel (75 mg per day) plus aspirin (100 mg per day) or aspirin alone (100 mg per day) between November 2004 and April 2011 (detailed profile included in Additional file 1). We excluded patients who had received other antiplatelet drugs, anticoagulants or thrombolytics during the study period. We also excluded patients who had severe comorbid conditions: increased risk of bleeding including diagnosis of severe hepatic insufficiency, renal failure or current peptic ulceration, history of systemic bleeding, other history of bleeding diathesis or coagulopathy, or a contraindication to aspirin or clopidogrel during the study period. Consequently, we identified 159 new users of clopidogrel plus aspirin and 834 new users of aspirin alone. The ethics committee of Nihon University School of Medicine approved the study protocol.

### Exposure and outcome

In this study, the index date was defined as the date of first prescription of the study drugs. The baseline measurement period (non-exposure period) was defined as within six months before the index date in the clopidogrel plus aspirin and aspirin alone cohorts. The exposure period (outcome measurement period) was defined as between two weeks and two months after the start of treatment with clopidogrel plus aspirin or aspirin alone to evaluate the short-term effect of the study drugs. Blood test data (creatinine, AST, and ALT levels, and hematological parameters including RBC count, WBC count, platelet count, hemoglobin level and hematocrit) were collected for each individual at the date nearest the index date in the baseline period, and at the date nearest two months after the start of treatment in the exposure period. Consequently, the mean (95% confidence interval (CI)) exposure of clopidogrel plus aspirin and aspirin alone users was 36.0 (33.8-38.2) days and 36.7 (35.8-37.7) days, respectively. There was no statistically significant difference in mean exposure days between them. Information on bleeding episodes of patients who were newly diagnosed with intracranial hemorrhage or gastrointestinal (GI) bleeding in the exposure period was collected.

### Covariates

For each individual, information on patient demographics (age and sex), medical history, current medication, and laboratory results was collected. Medical history included information on cerebrovascular disease (ICD-10 codes, I60-I69), ischemic heart disease (I20-I25), other heart disease (I30-I52), other peripheral vascular disease (I73), liver disease (K70-K77), kidney disease (N00-N19), gout (M10), thyroid gland disorders (E00-E07), hyperlipidemia (E78.0-E78.5), hypertension (I10), and diabetes mellitus (E10-E14) that had been diagnosed prior to the index date. We recorded current users of medication including antihypertensive agents, steroids, lipid-lowering drugs, insulin, oral antihyperglycemic agents, non-steroidal anti-inflammatory drugs (NSAIDs), proton pump inhibitors (PPIs), histamine2-receptor antagonists (H2 blockers), other anti-peptic ulcer agents, immunosuppressive drugs, diuretics, and anti-arrhythmic drugs, defined as patients who had received these agents in the 60 days preceding the index date.

### Statistical analysis

All analyses were performed with SAS software, version 9.2 (SAS Institute, Cary, NC). All reported p-values are two sided. A result was considered statistically significant if the p value was less than 0.05. To compare differences in baseline characteristics, we used *t*-test for continuous variables and chi-squared test for categorical data. Also, we used *t*-test to compare the mean values of laboratory parameters at baseline between clopidogrel plus aspirin users and aspirin alone users. The general linear model approach was performed to calculate multivariate-adjusted values of blood test parameters and to compare the adjusted least-squares means of changes from the baseline value to the exposure value between clopidogrel plus aspirin users and aspirin alone users. To reduce bias by controlling for baseline covariates between settings, two adjusted models are presented. The first model had regression (covariance) adjustment with the propensity score. This method is an effective tool to reduce bias in nonrandomized studies [21,22], and is described elsewhere [23]. In brief, the propensity score for each subject was obtained by fitting a logistic regression model that includes the predictor variable (i.e., clopidogrel plus aspirin users or aspirin alone users) as an outcome and all baseline covariates including age, sex, comorbid diseases (cerebrovascular disease, ischemic heart disease, other heart disease, liver disease, kidney disease, gout, thyroid gland disorders, hyperlipidemia, hypertension, and diabetes mellitus) and previous drugs (including antihypertensive agents, steroids, lipid-lowering drugs, oral antihyperglycemic agents, NSAIDs, PPIs, H2 blockers, other anti-peptic ulcer agents, immunosuppressive drugs, anti-arrhythmic drugs and chemotherapeutic drugs), as listed in Table 1. With the propensity score included in the general linear model, we assessed and compared the adjusted least-squares means of changes in laboratory parameters during the exposure period from baseline between clopidogrel plus aspirin users and aspirin alone users. The second model adjusted for age, sex and the remaining covariates, which were selected using a backward stepwise elimination method (p < 0.10). To estimate the relative risk of intracranial hemorrhage or GI bleeding for clopidogrel plus aspirin users and aspirin alone users, we used the odds ratio and 95% CI from logistic regression in which we controlled for covariates using the two adjustment models described above.

**Table 1:** Baseline characteristics of study population

**Characteristics**	**Clopidogrel plus aspirin**	**Aspirin alone**	**p**
	**(n = 159)**	**(n = 834)**	
Age (years, mean ± SE)	64.6 ± 1.0	68.3 ± 0.4	0.0005
Women	32 (20.13%)	329 (39.45%)	<0.0001
**Medical history**			
Cerebrovascular disease	40 (25.16%)	375 (44.96%)	<0.0001
Ischemic heart disease	140 (88.05%)	536 (64.27%)	<0.0001
Liver disease	38 (23.9%)	369 (44.24%)	<0.0001
Kidney disease	57 (35.85%)	378 (45.32%)	0.0273
Hypertension	107 (67.3%)	495 (59.35%)	0.0603
Diabetes mellitus	122 (76.73%)	606 (72.66%)	0.2879
Hyperlipidemia	147 (92.45%)	668 (80.10%)	0.0002
**Current medication**			
Insulin	3 (1.89%)	60 (7.19%)	0.0119
Oral hypoglycemic drug	25 (15.72%)	106 (12.71%)	0.3035
Lipid-lowering drug	38 (23.9%)	144 (17.27%)	0.0476
Antihypertensive drug	51 (32.08%)	590 (70.74%)	<0.0001
ARB	27 (16.98%)	199 (23.86%)	0.0579
ACEI	4 (2.52%)	49 (5.88%)	0.0841
Beta-blocker	7 (4.4%)	53 (6.35%)	0.3437
CCB	39 (24.53%)	524 (62.83%)	<0.0001
Thiazide diuretic	9 (5.66%)	127 (15.23%)	0.0013
Other	14 (8.81%)	80 (9.59%)	0.756
NSAID	12 (7.55%)	191 (22.9%)	<0.0001
Steroid	4 (2.52%)	82 (9.83%)	0.0026
H2 blocker	17 (10.69%)	455 (54.56%)	<0.0001
Proton pump inhibitor	19 (11.95%)	151 (18.11%)	0.059
Antiepileptic drug	2 (1.26%)	55 (6.59%)	0.008
Immunosuppressive drug	0 (0%)	18 (2.16%)	0.0616
Diuretic	8 (5.03%)	129 (15.47%)	0.0005
Antiarrhythmic drug	12 (7.55%)	104 (12.47%)	0.0765

Data are numbers of individuals (%) unless otherwise stated. The item of other peripheral vascular disease is not presented because its number was zero. Abbreviations: *ARB* angiotensin II receptor blocker, *ACEI* angiotensin-converting enzyme inhibitor, *CCB* calcium channel blocker, *NSAID* non-steroidal anti-inflammatory drug, *H2 blocker* histamine2-receptor antagonist.

## Results

The study included 159 patients who had been newly treated with clopidogrel plus aspirin and 834 patients who had been newly treated with aspirin alone. Table 1 shows the baseline characteristics of the patients. In clopidogrel plus aspirin users, the mean age was 64.6 years and 20.1 percent were women. Aspirin alone users were older and were more likely to be women than clopidogrel plus aspirin users; the mean age was 68.3 years and 39.5 percent were women. More than two-thirds of each cohort had ischemic heart disease, hyperlipidemia or diabetes mellitus, suggesting raised risk of cardiovascular disease. Clopidogrel plus aspirin users were more likely to have ischemic heart disease and hyperlipidemia, and were less likely to have cerebrovascular disease, liver disease, and kidney disease than aspirin alone users. In current medications, clopidogrel plus aspirin users were more likely to utilize lipid-lowering drugs than aspirin alone users. On the other hand, aspirin alone users were more likely to utilize calcium channel blockers, thiazide diuretics, NSAIDs, H2 blockers, diuretics and anti-arrhythmic drugs. Table 2 shows the mean values in laboratory parameters at baseline. The mean hemoglobin level in clopidogrel plus aspirin users was higher than that in aspirin alone users. None of the other tests showed any statistically significant difference in mean values at baseline between clopidogrel plus aspirin users and aspirin alone users. Because differences in baseline covariates, including age, sex, comorbid diseases and current medication, between clopidogrel plus aspirin users and aspirin alone users may create potential bias, we used a multivariate regression model and regression adjustment with propensity score to control for potential confounding covariates in our observational study.

**Table 2:** Baseline laboratory parameters of studied patients

**Laboratory test**	**Clopidogrel plus aspirin (n = 159)**	**Aspirin alone (n = 834)**	**p**
	**mean (95% CI)**	**mean (95% CI)**	
Creatinine (mg/dl)	0.93 (0.72, 1.14)	1.12 (1.03, 1.22)	0.0853
ALT (U/L)	35.33 (33.15, 37.52)	36.47 (35.52, 37.42)	0.3490
AST (U/L)	35.14 (32.96, 37.32)	36.56 (35.61, 37.51)	0.2409
WBC (10^3^/μL)	6.96 (6.30, 7.61)	6.99 (6.74, 7.25)	0.9210
RBC (10^6^/μL)	4.16 (4.06, 4.270)	4.07 (4.02, 4.12)	0.1177
PLT (10^3^/μL)	222.5 (205.3, 239.6)	239.9 (232.4, 247.4)	0.0675
Hemoglobin (g/dL)	13.14 (12.81, 13.47)	12.76 (12.61, 12.90)	0.0369
Hematocrit (%)	38.84 (37.89, 39.80)	37.87 (37.45, 38.29)	0.0664

Abbreviations: *ALT* alanine aminotransferase, *AST* asparate aminotransferase, *WBC* white blood cell count, *RBC* red blood cell count, *PLT* platelet count, *CI* confidence interval.

Table 3 shows the mean changes in WBC count during the exposure period compared with the baseline period. In clopidogrel plus aspirin users, the reduction of WBC count was significantly greater than that in aspirin alone users before and after adjustment for covariates. The mean changes in other laboratory parameters were not significantly different in clopidogrel plus aspirin users in comparison to those in aspirin alone users (data are included in Additional file 2). Table 4 shows the prevalence of patients who had hemorrhagic events during the exposure period. The rate of GI bleeding was 3.14 percent in clopidogrel plus aspirin users, as compared with 0.67 percent in aspirin alone users, with a relative risk of 2.61 (95% CI, 1.18 to 5.80; p = 0.0184) for propensity adjustment and a relative risk of 2.06 (95% CI, 1.02 to 4.13; p = 0.043) for the multivariate model, suggesting that the risk of GI bleeding was increased in patients treated with the combination of clopidogrel and aspirin. The risk of intracranial hemorrhage was not significantly different between clopidogrel plus aspirin users and aspirin alone users.

**Table 3:** Mean changes in laboratory test values during exposure period from baseline

**Laboratory test**	**Clopidogrel plus aspirin (n = 159)**	**Aspirin alone (n = 834)**	**p**
	**mean (95% CI)**	**mean (95% CI)**	
Δ** WBC (10**^**3**^**/μL)**			
Unadjusted	−1.65 (−2.099, -1.202)	−0.455 (−0.651, -0.259)	<0.0001*
Propensity adjustment	−1.509 (−1.999, -1.018)	−0.312 (−0.803, 0.179)	0.0008*
Multivariate model	−1.832 (−2.317, -1.346)	−0.435 (−0.634, 0.237)	<0.0001*

Δ indicates mean change in laboratory test value during exposure period from baseline. Data that showed a significant difference are presented. Abbreviations: *WBC* white blood cell count, *CI* confidence interval. *: p < 0.05 (aspirin plus clopidogrel vs aspirin alone).

**Table 4:** Prevalence of hemorrhagic events

**Hemorrhagic event**	**Clopidogrel plus aspirin (n = 159)**	**Aspirin alone (n = 834)**	**Propensity adjustment**		**Multivariate model**	
	**no. (%)**		**Relative risk (95% CI)**	**P**	**Relative risk (95% CI)**	**P**
Intracranial hemorrhage	5 (3.14%)	17 (2.04%)	1.71 (0.99, 2.94)	0.0507	1.23 (0.76, 2.01)	0.4009
Gastrointestinal bleeding	5 (3.14%)	6 (0.72%)	2.61 (1.18, 5.80)	0.0184	2.06 (1.02, 4.13)	0.0430

Abbreviation: *CI* confidence interval.

## Discussion

In this study, we evaluated and compared the effects of combination therapy of clopidogrel plus aspirin and aspirin monotherapy on laboratory parameters including creatinine, AST, ALT, hemoglobin level, hematocrit, WBC, RBC and PLT counts in a short-term administration period up to two months. We found that the reduction of WBC count in clopidogrel plus aspirin users was significantly greater than that in aspirin alone users. These results suggest that the hematological adverse effect on leukocytes is greater with combination therapy of clopidogrel plus aspirin than with aspirin monotherapy.

A variety of hematological adverse reactions, including leukopenia, agranulocytosis, and thrombocytopenia, have been reported in patients receiving clopidogrel or aspirin [24-27]. In the CAPRIE trial, the numbers of patients with a significant reduction in neutrophils were 0.10 percent and 0.17 percent in the clopidogrel and aspirin groups, respectively [24]. In this study, the decrease in mean WBC count during the exposure period from baseline was significant both in clopidogrel plus aspirin users and in aspirin alone users (data are included in Additional file 3). Our findings support these previous studies suggesting that the use of these antiplatelet agents may be associated with leukopenia. Furthermore, our findings suggested the possibility that the addition of clopidogrel to aspirin may enhance the adverse effect of aspirin alone on WBC count, although the mechanism of myelotoxicity of these antiplatelet agents is not clear. Our study is expected to help physicians make decisions on drug selection because the adverse effect of an antiplatelet therapy on leukocytes may be of clinical concern, especially for patients with borderline low WBC count.

The nature of antiplatelet therapy involves an inherent risk of bleeding complications. Some trials reported that the addition of aspirin to clopidogrel increases the risk of hemorrhage [6,11-13]. Our study showed that GI bleeding occurred more frequently in patients receiving clopidogrel plus aspirin than in patients receiving aspirin alone. Although patients who had increased risk of bleeding, including diagnosis of severe hepatic insufficiency, renal failure, current peptic ulceration or history of systemic bleeding, were excluded from the study population, there was a possibility that some covariates at baseline might impact on the results of hemorrhagic events. For instance, the risk of GI bleeding is increased by administration of NSAIDs, advanced age and liver disease, but is reduced by administration of PPI and H2 blockers. In this study, we used two adjustment methods to control for these potential confounding covariates, and thereby found that the risk of GI bleeding was increased with combination therapy of clopidogrel and aspirin compared with aspirin alone, with a relative risk ranging from 2.06 with the multivariate model and 2.61 for propensity adjustment. Our results were similar to the results of two large-scale randomized clinical trials reporting that dual antiplatelet therapy with clopidogrel and aspirin increased the risk of GI bleeding approximately 2-fold compared with aspirin alone [6,13].

Our study has several limitations. It was a retrospective observational study, which has some issues with respect to the potential for selection bias and confounding factors. However, these problems caused by non-randomized data could be solved by combination with robust statistics; for example, propensity score method [21]. We used rigorous statistical methods to control for potential confounding variables between clopidogrel plus aspirin and aspirin alone users, including propensity adjustment and a multivariate regression model. However, their ability to control for differences was limited to variables that were available or measurable. Second, this study was an investigation to compare the effects of clopidogrel plus aspirin and aspirin alone on laboratory tests focusing on a short-term administration period up to two months, but was not a long-term study that analyzed longitudinal data, including repeated measures of laboratory parameters. Whether the duration of treatment (especially, long-term treatment) is associated with the outcome is of interest because long-term maintenance of dual therapy (for about one year) is reasonable in patients with ST-elevation myocardial infarction according to the ACC/AHA guidelines [1]. We will examine this theme in our next study. Third, we did fix the daily dosage in both treatment groups: clopidogrel (75 mg per day) plus aspirin (100 mg per day) users and aspirin alone users (100 mg per day), because these dosages are typically widely used to initiate antiplatelet therapy. This study was not designed to assess the effects of clopidogrel and aspirin at each dosage, because it is difficult to determine whether or not pharmacodynamics is dose-dependent in clinical settings. Furthermore, this study was undertaken to compare the effects of clopidogrel plus aspirin and aspirin alone on laboratory test results in a Japanese population; the cohorts identified for the study included only Japanese patients. There are genetic polymorphisms in several CYP450 enzymes involved in the metabolism of clopidogrel, such as variants in CYP2C19, particularly CYP2C19*2, which are associated with variability in clopidogrel active metabolite bioavailability, antiplatelet effects, and clinical outcomes [28,29]. Because the frequency of genetic variability differs among ethnic groups, it cannot be concluded whether the present findings can be extended to people of other races. The findings of our study, based on a non-randomized design, call for further studies, such as similar analyses of larger international databases or randomized clinical trials for confirmation.

## Conclusion

Our study showed that the reduction of WBC count in clopidogrel plus aspirin users was significantly greater than that in aspirin alone users, and that the combination of clopidogrel and aspirin increased the risk of GI bleeding compared with aspirin alone. Our findings suggest that hematological adverse effects are greater with combination therapy of clopidogrel plus aspirin than with aspirin monotherapy, and support the experience noted in clinical practice that the use of dual antiplatelet therapy requires regular checks of hematological parameters.

## Abbreviations

ACEI: Angiotensin-converting enzyme inhibitor; ALT: Alanine aminotransferase; ARB: Angiotensin II receptor blocker; AST: Aspartate aminotransferase; CCB: Calcium channel blocker; CDW: Clinical Data Warehouse; CI: Confidence interval; GI: Gastrointestinal; H2 blocker: Histamine2-receptor antagonist; NSAID: Non-steroidal anti-inflammatory drug; NUSM: Nihon University School of Medicine; PLT: Platelet; PPI: Proton pump inhibitor; RBC: Red blood cell; TIA: Transient ischemic attack; WBC: White blood cell.

## Competing interests

The authors declare that they have no competing interest.

## Authors’ contributions

YT conceived the study, participated in its design and drafted the manuscript. YN performed the statistical analyses. YT, TN and SA interpreted the data. All authors have read and approved the final manuscript.

## Supplementary Material

Additional file 1 Identification of study population. Click here for file

Additional file 2 Mean changes in laboratory test values during exposure period from baseline. Click here for file

Additional file 3 Unadjusted and adjusted mean (95% CI) laboratory test values in clopidogrel plus aspirin users and aspirin alone users. Click here for file
