# Supplementary material for: Comparative effect of clopidogrel and aspirin versus aspirin alone on laboratory parameters: a retrospective, observational, cohort study
Source: Cardiovasc Diabetol. 2013 Jun 14;12:87. doi: 10.1186/1475-2840-12-87 (PMC3687565; doi:10.1186/1475-2840-12-87)
Supplement: Additional file 1 — Identification of study population. [file 1475-2840-12-87-S1.docx]

**Additional file 1:** Identification of study population.

#1) Inclusion criteria: Japanese patients aged over 20 years who had been newly treated with clopidogrel (75 mg per day) plus aspirin (100 mg per day) or aspirin alone (100 mg per day) between November 2004 and April 2011 were identified for the study. We identified 1244 patients treated with clopidogrel plus aspirin and 9624 patients treated with aspirin alone who fulfilled the above criteria.

#2) Exclusion criterion 1: We excluded patients who had received other antiplatelet drugs, anticoagulants or thrombolytics during the study period.

#3) Exclusion criterion 2 (handling missing data): Blood test data were collected for each individual at the date nearest the start of treatment in the baseline period, and at the date nearest two months after the start of treatment in the exposure period. The baseline period was defined as within six months before the start of treatment. The exposure period was defined as between two weeks and two months after the start of treatment. If patients had a bleeding event during the exposure period, data were collected at the date before its occurrence. If patients had been diagnosed with acute myocardial infarction during the study period, data were collected at a date excluding the two-week period after its occurrence. We excluded patients who had missing data in either the baseline or exposure period for any laboratory parameter. We also excluded patients who had outliers defined as values outside mean ± 3 standard deviations for any laboratory parameter.

#4) Exclusion criterion 3: We excluded patients who had severe comorbid conditions: increased risk of bleeding including diagnosis of severe hepatic insufficiency, renal failure or current peptic ulceration, history of systemic bleeding, other history of bleeding diathesis or coagulopathy, or a contraindication to aspirin or clopidogrel during the study period.
