# Supplementary material for: Comparative effect of clopidogrel and aspirin versus aspirin alone on laboratory parameters: a retrospective, observational, cohort study
Source: Cardiovasc Diabetol. 2013 Jun 14;12:87. doi: 10.1186/1475-2840-12-87 (PMC3687565; doi:10.1186/1475-2840-12-87)
Supplement: Additional file 2 — Mean changes in laboratory test values during exposure period from baseline. [file 1475-2840-12-87-S2.docx]

**Additional file 2:** Unadjusted and adjusted mean (95% CI) laboratory test values in clopidogrel plus aspirin users and aspirin alone users.

| Laboratory test | | Clopidogrel plus aspirin (n=159) | | | | | | |
| --- | --- | --- | --- | --- | --- | --- | --- | --- |
|  |  | Unadjusted | | |  | Adjusted † | | |
|  |  | Mean | (95%CI) | p-value |  | Mean | (95%CI) | p-value |
| WBC (10^3^/μL) | |  |  |  |  |  |  |  |
|  | baseline | 7.74 | (7.34, 8.14) | <.0001* |  | 7.99 | (6.62, 9.36) | <.0001* |
|  | exposure | 6.09 | (5.69, 6.49) |  |  | 6.34 | (4.97, 7.71) |  |
|  | |  | | | | | | |
| Laboratory test | | Aspirin alone (n=834) | | | | | | |
|  |  | Unadjusted | | |  | Adjusted † | | |
|  |  | Mean | (95%CI) | p -value |  | Mean | (95%CI) | p -value |
| WBC (10^3^/μL) | |  |  |  |  |  |  |  |
|  | baseline | 7.24 | (7.05, 7.42) | 0.0009* |  | 7.35 | (6.88, 7.81) | <.0001* |
|  | exposure | 6.78 | (6.59, 6.97) |  |  | 6.70 | (6.24, 7.16) |  |

Abbreviations: WBC, white blood cell count; CI, confidence interval. *: p<0.05 (baseline vs exposure). † Analyses were adjusted for age, sex and the remaining other covariates, which were selected using a backward stepwise elimination method.
