# Supplementary material for: Comparative effect of clopidogrel and aspirin versus aspirin alone on laboratory parameters: a retrospective, observational, cohort study
Source: Cardiovasc Diabetol. 2013 Jun 14;12:87. doi: 10.1186/1475-2840-12-87 (PMC3687565; doi:10.1186/1475-2840-12-87)
Supplement: Additional file 3 — Unadjusted and adjusted mean (95% CI) laboratory test values in clopidogrel plus aspirin users and aspirin alone users. [file 1475-2840-12-87-S3.docx]

Additional file 3: Mean changes in laboratory test values during exposure period from baseline.

| Laboratory Test | Clopidogrel plus aspirin (n=159) |  | Aspirin alone (n=834) | p |
| --- | --- | --- | --- | --- |
|  | mean (95% CI) |  | mean (95% CI) |  |
| **Δ Creatinine (mg/dl)** |  |  |  |  |
| Unadjusted | 0.022 (-0.039, 0.082) |  | -0.025 (-0.052, 0.001) | 0.1617 |
| Propensity adjustment | 0.013 (-0.075, 0.100) |  | -0.019 (-0.106, 0.069) | 0.615 |
| Multivariate model | 0.033 (-0.032, 0.099) |  | -0.025 (-0.052, 0.002) | 0.1047 |
| **Δ ALT (U/L)** |  |  |  |  |
| Unadjusted | 0.10 (-9.97, 10.17) |  | -5.15 (-9.55, -0.76) | 0.3482 |
| Propensity adjustment | 0.72 (-11.33, 12.77) |  | -5.27 (-9.85, -0.70) | 0.3849 |
| Multivariate model | -0.22 (-11.34, 10.90) |  | -2.96 (-9.82, 3.91) | 0.6315 |
| **Δ AST (U/L)** |  |  |  |  |
| Unadjusted | -14.69 (-31.11, 1.74) |  | -9.17 (-16.34, -2.00) | 0.5459 |
| Propensity adjustment | -13.03 (-32.69, 6.63) |  | -9.48 (-16.95, -2.02) | 0.7523 |
| Multivariate model | -15.35 (-36.01, 5.30) |  | -7.31 (-20.63, 6.01) | 0.4267 |
| **Δ WBC (10^3^/μL)** |  |  |  |  |
| Unadjusted | -1.65 (-2.099, -1.202) |  | -0.455 (-0.651, -0.259) | <0.0001* |
| Propensity adjustment | -1.509 (-1.999, -1.018) |  | -0.312 (-0.803, 0.179) | 0.0008* |
| Multivariate model | -1.832 (-2.317, -1.346) |  | -0.435 (-0.634, 0.237) | <0.0001* |
| **Δ RBC (10^6^/μL)** |  |  |  |  |
| Unadjusted | -0.065 (-0.135, 0.005) |  | -0.063 (-0.091, -0.033) | 0.9709 |
| Propensity adjustment | -0.132 (-0.221, -0.042) |  | -0.051 (-0.083, -0.018) | 0.1176 |
| Multivariate model | -0.108 ( -0.186, -0.029) |  | -0.046 ( -0.082, -0.009) | 0.1538 |
| **Δ PLT (10^3^/μL)** |  |  |  |  |
| Unadjusted | 3.01 (-11.48, 17.50) |  | -5.36 (-11.69, 0.97) | 0.299 |
| Propensity adjustment | 3.45 (-9.19, 16.09) |  | -9.94 (-22.6, 2.71) | 0.1416 |
| Multivariate model | 3.58 (-19.89, 27.05) |  | -5.53 (-24.64, 13.58) | 0.2893 |
| **Δ Hemoglobin (g/dL)** |  |  |  |  |
| Unadjusted | -0.207 (-0.427, 0.012) |  | -0.175 (-0.271, -0.079) | 0.7949 |
| Propensity adjustment | -3.157 (-21.65, 15.34) |  | -4.184 (-10.88, 2.511) | 0.9233 |
| Multivariate model | -0.311 (-0.557, -0.066) |  | -0.116 (-0.228, -0.003) | 0.1503 |
| **Δ Hematocrit (%)** |  |  |  |  |
| Unadjusted | -0.642 (-1.291, 0.007) |  | -0.533 (-0.817, -0.250) | 0.7632 |
| Propensity adjustment | -0.411 (-0.690, -0.131) |  | -0.136 (-0.238, -0.035) | 0.089 |
| Multivariate model | -0.634 (-1.314, 0.046) |  | -0.533 (-0.828, -0.239) | 0.7843 |

Δ indicates mean change in laboratory test value during exposure period from baseline. Abbreviations: ALT, alanine aminotransferase; AST, asparate aminotransferase; WBC, white blood cell count; RBC, red blood cell count; PLT, platelet count; CI, confidence interval. *: p<0.05 (aspirin plus clopidogrel vs aspirin alone).
